# Mechanics Predicts Effective Critical-Size Bone Regeneration Using 3D-Printed Bioceramic Scaffolds

**DOI:** 10.1007/s13770-023-00577-2

**Published:** 2023-08-22

**Authors:** Pablo Blázquez-Carmona, Juan Mora-Macías, Francisco J. Martínez-Vázquez, Juan Morgaz, Jaime Domínguez, Esther Reina-Romo

**Affiliations:** 1 Escuela Técnica Superior de Ingeniería, Universidad de Sevilla, Avenida Camino de los Descubrimientos s/n, 41092 Seville, Spain; 2 Escuela Técnica Superior de Ingeniería, Universidad de Huelva, Huelva, Spain; 3 Departamento Medicina y Cirugía Animal, Universidad de Córdoba, Campus Universitario de Rabanales, Córdoba, Spain; 4 Instituto de Biomedicina de Sevilla (IBiS), Universidad de Sevilla, Seville, Spain

**Keywords:** Tissue engineering, Bioceramic scaffold, Mechanobiology, Computerized tomography, Bone mineral density

## Abstract

**BACKGROUND:**

3D-printed bioceramic scaffolds have gained popularity due to their controlled microarchitecture and their proven biocompatibility. However, their high brittleness makes their surgical implementation complex for weight-bearing bone treatments. Thus, they would require difficult-to-instrument rigid internal fixations that limit a rigorous evaluation of the regeneration progress through the analysis of mechanic-structural parameters.

**METHODS:**

We investigated the compatibility of flexible fixations with fragile ceramic implants, and if mechanical monitoring techniques are applicable to bone tissue engineering applications. Tissue engineering experiments were performed on 8 ovine metatarsi. A 15 mm bone segment was directly replaced with a hydroxyapatite scaffold and stabilized by an instrumented Ilizarov-type external fixator. Several *in vivo* monitoring techniques were employed to assess the mechanical and structural progress of the tissue.

**RESULTS:**

The applied surgical protocol succeeded in combining external fixators and subject-specific bioceramic scaffolds without causing fatal fractures of the implant due to stress concentrator. The bearing capacity of the treated limb was initially altered, quantifying a 28–56% reduction of the ground reaction force, which gradually normalized during the consolidation phase. A faster recovery was reported in the bearing capacity, stiffening and bone mineral density of the callus. It acquired a predominant mechanical role over the fixator in the distribution of internal forces after one post-surgical month.

**CONCLUSION:**

The bioceramic scaffold significantly accelerated *in vivo* the bone formation compared to other traditional alternatives in the literature (e.g., distraction osteogenesis). In addition, the implemented assessment techniques allowed an accurate quantitative evaluation of the bone regeneration through mechanical and imaging parameters.

## Introduction

When skeletal tissues have been critically devastated or lost by congenital anomaly, poorly corrected trauma, osteomyelitis or cancer, the preferred procedures for acute care surgeons are distraction osteogenesis [1–4], massive or Papineau open cancellous bone grafting [5], and vascularized free fibula transference [6]. Despite reported regenerative effectiveness through optimization of its biomechanical factors (e.g., rate and frequency of distraction), the Ilizarov method is associated with high risks related to viscoelastic and structural changes in the surrounding soft tissues [7–9]. Regarding bone substitutes, autografts and allografts are currently the most implanted materials due to their unmatched osteoinductive, osteoconductive and biocompatible properties [10]. Nevertheless, the rising number of trauma patients with long bone defects is increasing the global demand [11]. To satisfy their insufficient availability, tissue engineering (TE) has rapidly emerged as the ultimate orthopaedical hope to unlimitedly replace previous natural approaches. They offer artificial linkage structures called scaffolds to promote osteogenesis while reducing other grafting shortcomings, including donor site complications and morbidity [12] or potential donor pathogen transmission [13]. Most recent investigations are focused on *in vitro* and *in silico* models trying to reach the ideal manufacturing technology, configurations, internal architecture and, indeed, the biomaterial substitute. Specifically, bioceramic materials, composed of partially or non-crystalline ceramics (e.g., hydroxyapatite, calcium phosphate cement or glass–ceramics), are gaining popularity in clinical research due to their demonstrated key mechanisms leading to enhanced new hard tissue growth by regulating osteoblast proliferation, differentiation, and gene expression [14]. Unlike commonly employed metallic materials that could inherently obstruct the invasion of blood vessel walls or require a periodic replacement [15], bioceramics allow a “leave nothing behind” approach through a gradual resorption as the new bone tissue grows. Moreover, bioceramic scaffolds are generally built through additive manufacturing technologies, which allow prototyping and comparing patient-specific scaffolds with different microarchitectures and controlled pore size distributions [16, 17]. So far, bioceramic scaffolds have been tested primarily on animal studies with limited success [18–21] and more *in vivo* investigations are needed.

During bone regeneration processes, continuous monitoring is essential in the early healing stages when rejection of grafts and implants or non-unions are more likely to occur. The traditional *in vivo* clinical evaluations, such as plain film radiology, clinical signs of inflammation or pain, and mobility examinations of the operated limb, are not accurate enough to ensure that a bone callus is forming properly or to make relevant decisions, including surgical reintervention [22–24]. In the last decades, engineers have developed newfangled tracking alternatives through the instrumentation of medical devices. Among these strategies are using acoustic emissions [25], vibrational tests analyzing the wave propagation through the tissue and its resonant frequency [26], or osteointegration monitoring using ultrasounds [27]. Nonetheless, these techniques are difficult to standardize or transfer to routine clinical use today due to their significant dependence on their experimental protocol and testing boundary conditions [28]. By cons, the instrumented external fixations helped control osteogenesis based on indirectly estimating apparent mechanical properties and loading capacity of the callus tissue from the force, stress, or raw strain data [1–4, 29–35]. This approach has been traditionally applied *in vivo* to distraction osteogenesis or fracture healing treatments, thus providing additional practical information: comparison of the effectiveness of different protocols or the optimization of the time-point for the fixator’s removal. For instance, Liu et al. [32] recently found that an axial load-share ratio below 10% is safe to disassemble a monolateral external fixator when treating human trauma. As far as the authors’ knowledge, no previous research has explored the applicability of these monitoring technologies or the mechano-structural evolution of the TE callus compared to other clinical alternatives (e.g., bone transport or bone grafting). In TE, they would be particularly interesting due to the limited imaging follow-up resulting from the high radiopacity of many scaffolding base materials and their typical length scale.

Unlike the above, the critical-size and load-bearing bone defects replaced by scaffolding are usually stabilized by means of internal splints in the literature [18–21]. Their stiffer assembly methods provide better bony alignment and rotation, more accurate control of interfragmentary movements and, consequently, less significant mechanical restrictions for the structural integrity of brittle host implants. For example, Vidal et al. [20] resorted to internal fixations when treating a 35 mm sheep metatarsal defect with calcium phosphate scaffolds. Despite the reported high damage-tolerant behavior of the biomorphic ceramic scaffolds’ hierarchical architecture, Kon et al*.* [18] also entrusted with stabilizing their metatarsal defects on direct screwing metallic fixation plaques. Furthermore, beyond the more feasible installation of measuring sensors, all of them forfeit the external approaches’ advantages of requiring less significant body invasion that benefits the patient by avoiding extensive soft tissue damage, reducing blood loss during surgeries, and simplifying the associated soft tissue management [36, 37]. All this leads to early mobilization of the patient and a shorter hospitalization [27]. Pobloth et al. [38] succeeded in combining a hybrid-ring external fixator with a tubular-shaped scaffold made of β‐TCP granules attached to a resorbable polymer template in an ovine tibia model. The certain deformability of its base material allowed a better dissipation of the mechanical forces *in vivo*. Oh et al. [39] designed an external fixator adaptable to different axial stiffnesses aimed at tissue engineering applications in rodent models, as far as the authors are concerned, its effectiveness has not been tested *in vivo*. Considering the previous technical difficulties, a safe surgical configuration and regenerating efficacy are still a huge challenge in implementing these ceramic scaffolds as a commercial product to treat human orthopedic defects under a mechanical environment controlled by a flexible external fixation.

Based on the previous background, the goals of this work are: (1) to establish a surgical procedure and an implantation protocol that allow solving the clinical challenge of combining subject-specific bioceramic scaffolds under a flexible mechanical environment; (2) to test for the first time methods of mechanical assessment of the regeneration process in tissue engineering and discuss its usefulness for clinical decision making; and (3) to discuss the recovery of the analyzed mechano-structural parameters in comparison with other alternative regeneration processes already monitored in the literature (e.g., distraction osteogenesis or fracture healing).

## Materials and methods

Tissue engineering surgeries were performed in the ipsilateral hind metatarsus of female Merino sheep aged between 3 and 5 years (*n* = 8). Specifically, 15 mm intermediate bone defects were regenerated aided by subject-specific bioceramic scaffolds stabilized with flexible fixations. Animal ethics of the University of Córdoba approved this study (2021PI/21). The welfare of the sheep was guaranteed during the complete surgical interventions and experimental phases following the HIPAA, European (2010/63/UE) and national (RD 1201/2005) norms on animal experimentation. As shown in Table 1, animals were slaughtered at different time-points of the consolidation phase so as to analyze the evolution of computed tomography (CT) parameters, whose regular appraisal was limited by the complex segmentation resulting from the reflections of the fixator's metallic components. The manufacturing process of the implants, the surgical intervention, and the set of *in vivo* and *ex vivo* experiments are specified as follows, including images in Fig. 2. Throughout the following subsections, fitting functions will be built to illustrate the trends of most of the parameters.

**Table 1:** Individual data of each specimen: consolidation days before euthanasia, time-points at which tomographic scans were performed, estimation of the apparent stiffness of the proximal (${K}_{b,p}$), distal (${K}_{b,d}$) of bone-fixator system, and equivalent cortical stiffness (${K}_{b}$)

Sheep	Days	CT time-points	${K}_{b,p}$[kN/mm]	${K}_{b,d}$[kN/mm]	${K}_{b}=\frac{{{K}_{b,d}\bullet K}_{b,d}}{{{K}_{b,d}+K}_{b,d}}$
S1	365	365, 285, 176	309.85	356.74	165.82
S2	219	219	144.51	138.36	70.68
S3	194	194, 158	199.86	206.13	101.47
S4	152	152	173.25	190.27	90.68
S5	123	123	236.96	333.16	138.13
S6	65	65	160.67	136.69	73.86
S7	51	51	206.71	219.49	106.45
S8	30	30	218.43	236.98	113.67

### Fabrication and preparation of the scaffold

**Fig. 1:**
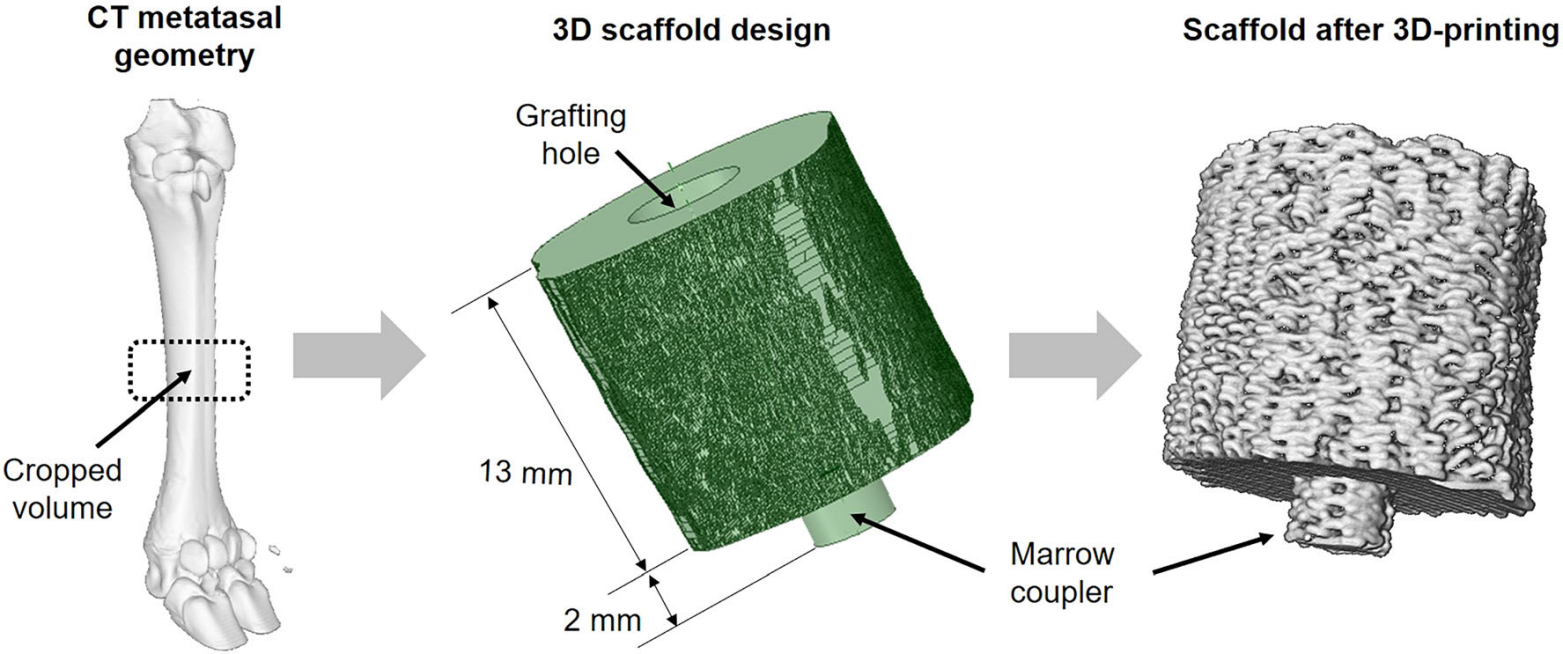
Design of the patient-specific outer scaffold geometry: (1) cropping an intermediate 13 mm bone segment from computed tomography images of each ovine metatarsus; and (2) addition of a cylindrical hole and a coupler to graft the structure with cancellous bone and to immobilize the implant inside the bone marrow, respectively

The subject-specific geometry of the scaffolds was shaped from tomography scans performed on the ovine metatarsi before surgery. DICOM images (voxel size 0.115 × 0.115 × 0.600 mm) were interactively thresholded and reconstructed using the open-source software InVesalius® (Renato Archer Information Technology Center, Amarais, Brazil). As shown in Fig. 1, an intermediate 13 mm bony fragment was cropped from the metatarsal 3D geometry. Then, the inner medullary cavity was filled. A cylindrical coupler (4 mm Ø; 2 mm height) was subsequently built in the 3D geometry at the bone marrow location of one end face to restrict the scaffold’s mobility within the defect (see Fig. 1). On the other end, a hole (4 mm Ø; 10 mm height) was created to be stuffed by grafting tissue during the surgery, thus stimulating the implant’s biological response. The inner architecture was numerically optimized in a previous work to maximize the osteoinductive and osteoconductive properties while ensuring the mechanical integrity under the physiological loads of this bone model [40]. Thereby, under the mechanical limits imposed by these physiological loads, the porosity, specific surface area and pore size were maximized to guarantee cell diffusion, adhesion and proliferation in the implant. The resulting microstructure presents 59.30% of porosity, 5768.91 m^−1^ of specific surface area, and 360.80 μm of pore size [40].

**Fig. 2:**
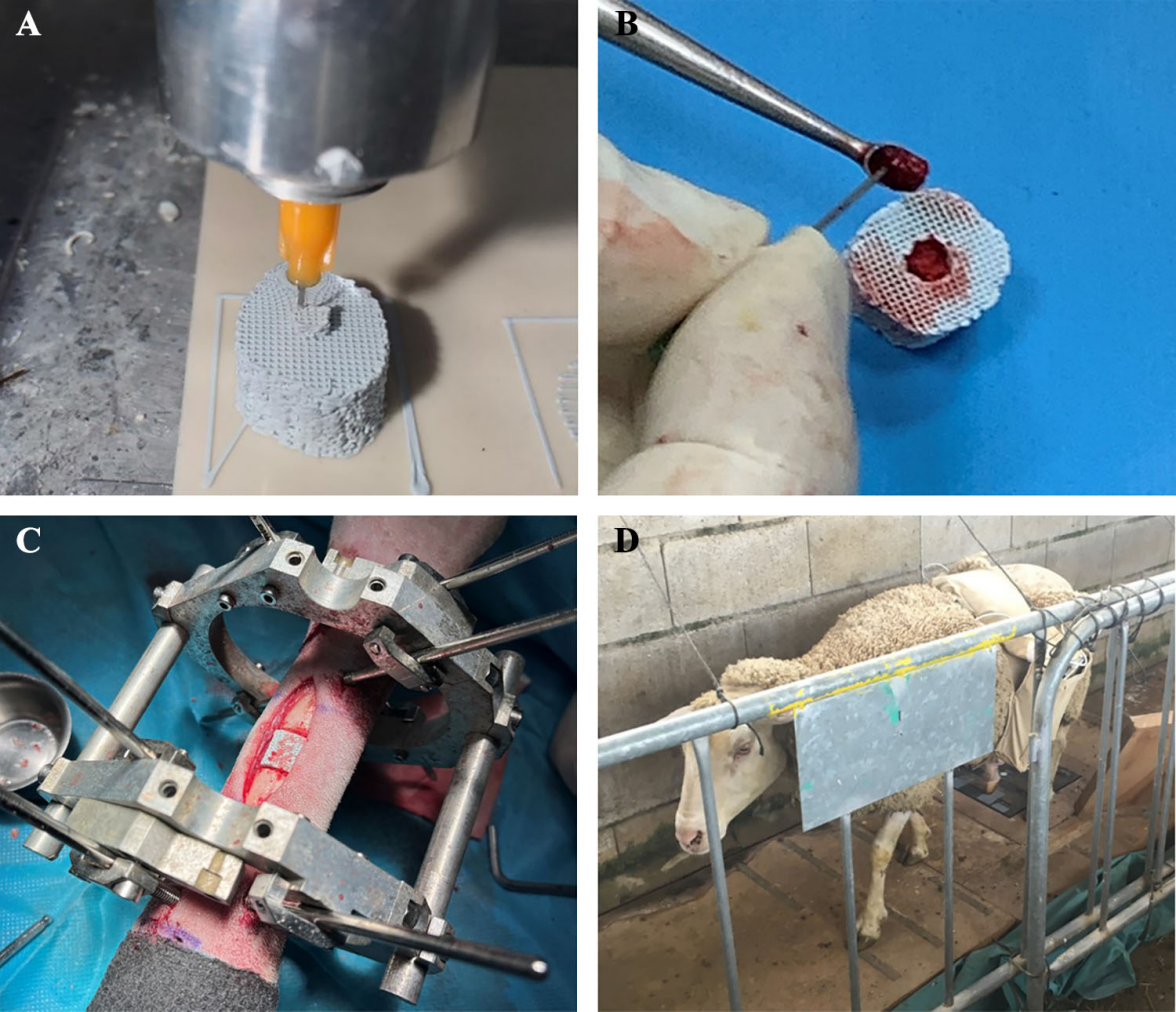
**A–D**: Protocol steps for our ovine tissue engineering study: **A** Manufacturing of the ceramic metatarsal scaffold via robocasting; **B** Scaffold filling with humerus cancellous autograft; **C** Implantation of the external fixator and replacement of the bone fragment by the biologically enhanced scaffold; **D** Routine gait experiments in the instrumented walking circuit

Implants were manufactured following a layer-wise long-pile pattern of rods, using robotic deposition device (3-D Inks Still-water®, Tulsa, Oklahoma, USA) and a clinically proven 45 vol. % hydroxyapatite (HA) ink, as shown in Fig. 2A. The selected base material mimics the bone chemical composition and morphology [41, 42]. Moreover, its biodegradability does not require surgical removal of the scaffold after the consolidation process. Once manufactured, the structures were dried at room temperature, heated at 400 °C for 1 h to eliminate organic components, and sintered at 1300 °C for 2 h. Implants were chemically sterilized under a high formaldehyde concentration at 60 °C and with a relative humidity of 75 to 100%.

### Surgical procedure

Sheep were initially immobilized. Antibioterapy and inflammation were pre-treated with Amoxiciline 15 mg/kg Clamoxyl® and Meloxicam 0.2 mg/kg Metacam®, respectively. Animals were sedated with detomidine 20 µg/kg Detogesic® and morphine 0.2 mg/kg Morfina B.Braun®. After 10 min, the sheep were positioned in sternal decubitus induction, and endotracheal intubation was carried out. General anesthesia was maintained with isoflurane IsoVet® to expiratory concentrations of 1–1.2%. The oxygen saturation, electrocardiogram, respiratory gases, body temperature, and blood pressure (catheterizing pedal or auricular arteries) were controlled during the surgery.

The surgeries consisted mainly of assembling an external fixator (stiffness ${K}_{f}$ of 593 N/mm) on the bone and substituting a fragment for the scaffold. An insight into this fixator, already implanted, is given in Fig. 2C. The fixation is an Ilizarov-type device composed of two circular aluminum frames fixed individually to the bone by six drilled 4 mm Ø Schanz pins, two bicortical and one unicortical per frame. The frames are additionally externally interconnected by bars instrumented with Burster® 8431–6001 load cells (Burster, Gernsbach, Germany), which tolerate bending moments while real-time measuring forces through the fixation. A previous study provides more details about the fixation design, as well as the *in vitro* and *in vivo* calibration of the wireless acquisition platform [43]. Once the bone was stabilized, two osteotomies were performed in the intermediate part of the metatarsus, removing the resulting 15 mm bone fragment. Thereupon, cancellous autograft was harvested from the lateral side of the humerus head. A 7 cm incision was performed with electrical scalping, and a drill was used to access the requested tissue. After filling the scaffold’s grafting cavity with part of the spongy tissue, the implant was finally inserted into the induced defect (see Fig. 2B, C). The scaffold coupler was fitted in the distal bone marrow. As a consequence of the length of the main scaffold body, a 1–2 mm gap remained at its proximal interface with the metatarsal fragment. This space was filled with the remaining cancellous tissue, thus connecting both surfaces and preventing unexpected stress concentrations in the metatarsal-implant contact that could fracture the structure. In addition, operated animals were recovered with oxygen under assistance. Post-operative analgesia was also provided (meloxicam Metacam® and opioids) according to pain scales in sheep.

### *In vivo* mechanical monitoring

**Fig. 3:**
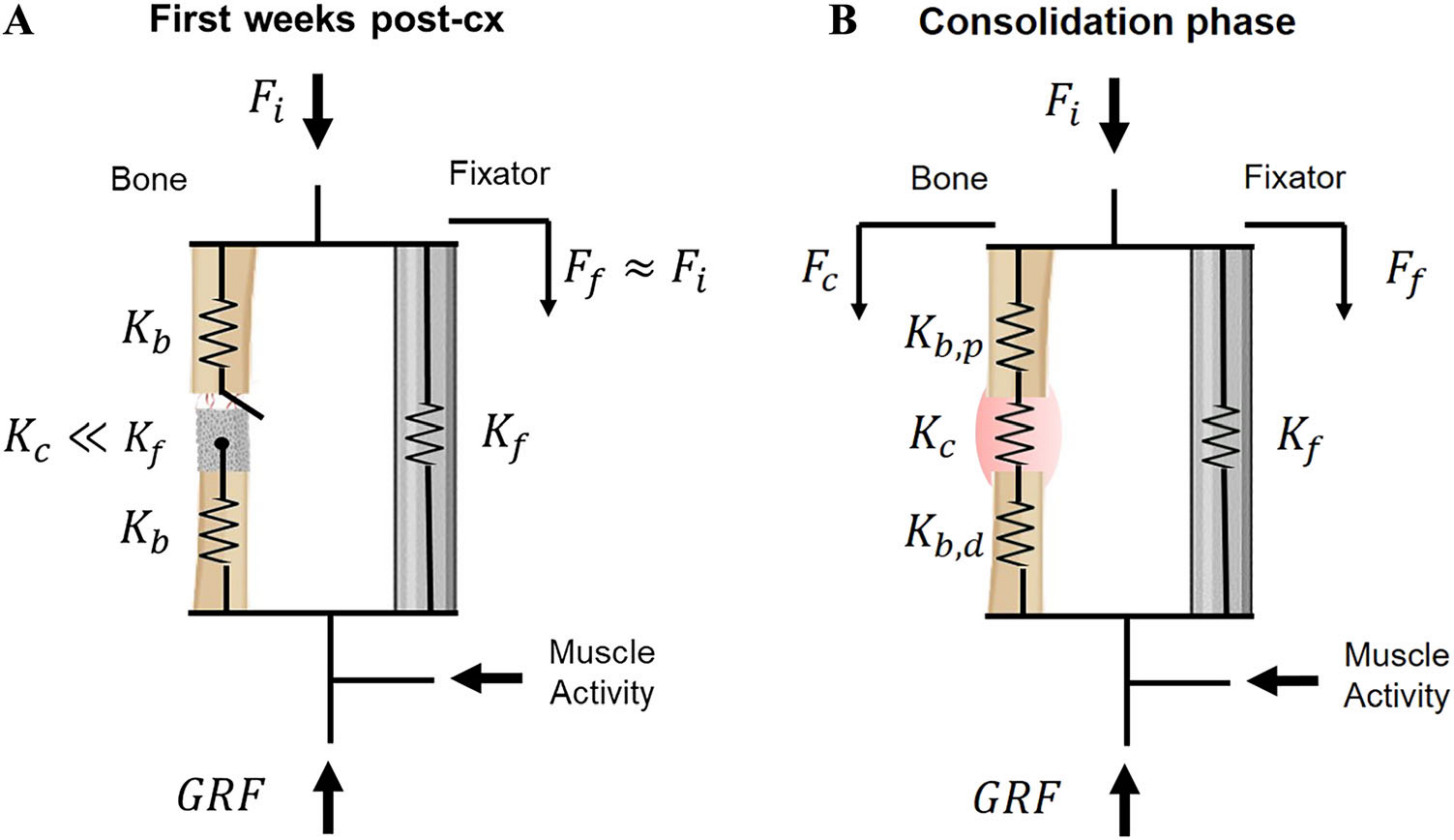
**A–B**: Mechanical models of the internal force ($${F}_{i}$$) distribution between the bone ($${F}_{C})$$ and the external fixator ($${F}_{f}$$) as a function of the ground reaction force ($$GFR$$), muscle activity, and the components’ stiffness: proximal and distal diaphysis fragments ($${K}_{b,p}$$ and $${K}_{b,d}$$), bone callus ($${K}_{C}$$), and fixation ($${K}_{f}$$). **A** Scheme during the first weeks after surgery without mature connection between the cortical bone and the scaffold; **B** Regular scheme contains the callus stiffness $${K}_{C}$$ during the consolidation phase

The evolution of several mechanical parameters involved in bone regeneration was assessed. After one latency week, stance analysis was performed two or three times weekly through walking tests in a circular gait circuit instrumented with a wireless load platform Pasco PS-2141® (PASCO, Roseville, CA, USA), as illustrated in Fig. 2D. The ground reaction curves of a minimum 8 treads at an amble speed (2–4 km/h) were captured in every experiment, thus analyzing the data as a daily average. This analysis focused on the maximum ground reaction force ($GRF$) due to its proven interrelationship with the repair of bone defects in other regeneration processes [30, 33]. Furthermore, the forces through the fixator during the analyzed animal's stance phases (${F}_{f}$) were simultaneously acquired using the sensors specified above. Following a similar strategy to previous works [9, 30, 44], the internal force through the metatarsus (${F}_{i}$) was estimated by assuming a constant ratio with the $GRF$. Using the bone-fixator mechanical system specified in Fig. 3A, this relationship was fixed during the early regeneration stage of every specimen, the first 2 post-surgical weeks, when there is hardly any mature bridging connection between the scaffold and the remaining fragments. This fact allows considering that the fixation loads are approximately equal to the internal force (${F}_{i}\approx {F}_{f}$). In the following consolidation phase, the evolution of the bone callus loading capacity (${F}_{C}$) was assessed according to Fig. 3B as:

1 $${F}_{C}={F}_{i}-{F}_{f}$$

Finally, the apparent callus stiffness ${K}_{c}$ was determined by assuming homogeneous mechanical properties in the tissue. From the bone-fixator model (Fig. 3B), these stiffness values were easily estimated from the ${F}_{i}$ distribution through the metatarsus, the stiffness of the external fixator ${K}_{f}$, and the apparent stiffness of the remaining proximal and distal metatarsal fragments within the fixation, ${K}_{b,p}$ and ${K}_{b,d}$. These cortical stiffness values differed from each other due to their geometrical interdifferences, and they were estimated from the CT images in the sacrifice specified in the following sections and supposing an elastic modulus of 21 GPa [9]. Their values per animal are also detailed in Table 1. Bearing in mind all this and considering an equivalent system displacement during the sheep’s thread in lines, the ${K}_{c}$ was estimated using Eq. 2:

2 $${K}_{c}={K}_{f}\cdot \frac{{F}_{c}\cdot {K}_{b}}{{F}_{f}\cdot {K}_{b}+{F}_{c}\cdot {K}_{f}}$$

where ${K}_{b}$ is the equivalent stiffness of ${K}_{b,p}$ and ${K}_{b,d}$ springs in line (see Fig. 3B). The mechanical analysis was limited to the *in vitro* calibration range of the measurement equipment with a low level of errors and uncertainties in the estimation of stiffness [43]. From that time-point, gait analysis continued to be performed less frequently to check the general tendency of these parameters.

### Imaging follow-up

**Fig. 4:**
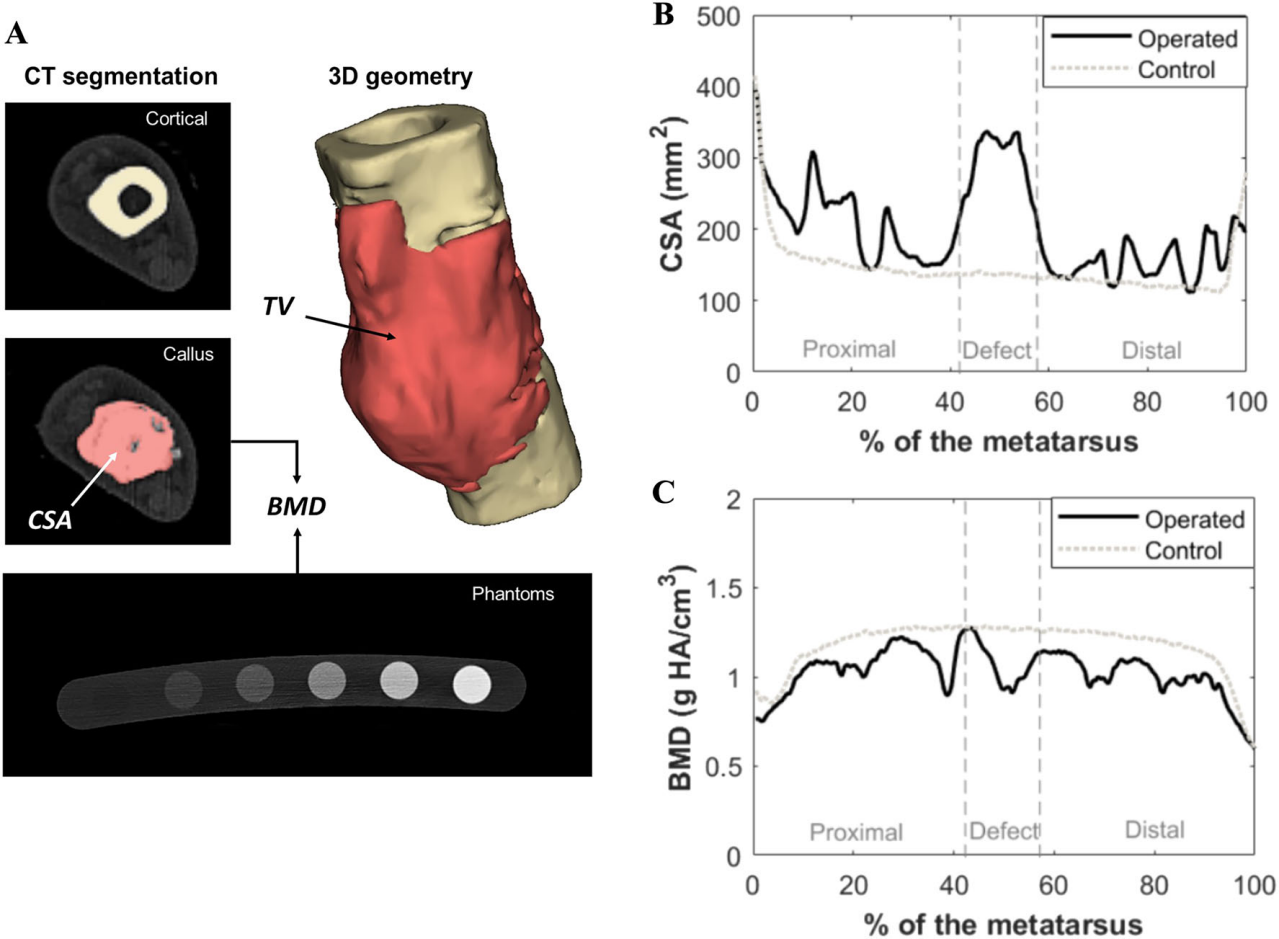
**A–C**: CT analysis of the operated limbs to analyze the geometry and the mineralization state of the bone callus at different time-points of the regeneration process. **A** Segmentation of the cortical, callus tissue and phantoms to further calculate the total volume (TV), cross-sectional area (CSA) and bone mineral density (BMD) evolution in the 3D generated geometry; **B** Example of the monitored evolution of the CSA along the treated and control metatarsus; **C** Example of the BMD evolution along the treated and control metatarsus. Peak values outside the defect limits are due to the woven bone formed around the pins of the external fixator

A radiographic follow-up was periodically performed to verify the regeneration progress through dorsoplanar and lateral views qualitatively. As previously stated, CT images of the treated limbs were additionally taken at the sacrifice time-points of the consolidation phase. Furthermore, the fixation was removed prior to sacrifice in some long-term specimens, enabling intermediate CT images. The non-operated contralateral limbs were included in the images and processed as control data. The resolution of the images was around 300 µm/voxel, and their manual segmentation was performed using the medical software InVesalius®. Figure 4A shows a scheme of the segmentation process and the generation of the three-dimensional geometry. The average callus cross-sectional area within the defect ($CSA$) and total callus volume ($TV$), including 1 cm of external tissue around the distal and proximal cortical fragment, were calculated. Finally, the apparent bone callus mineral density ($BMD$) was estimated as an indirect measure of the scaffold ossification. The phantoms QRM-BDC/6–200® (PTW, Freiburg, Germany) were used as a screening tool to correlate the mineral density (0–0.8 g HA/cm^3^) with the CT Hounsfield Units linearly. The contralateral limbs were simultaneously included in the scans as a control. An example of the monitored evolution of $CSA$ and $BMD$ along the axial metatarsal cross-section in one of the CT scans of the study is shown in Fig. 4B–C, including a comparison with the contralateral control limb.

## Results

### Gait analysis and mechanical healing

**Fig. 5:**
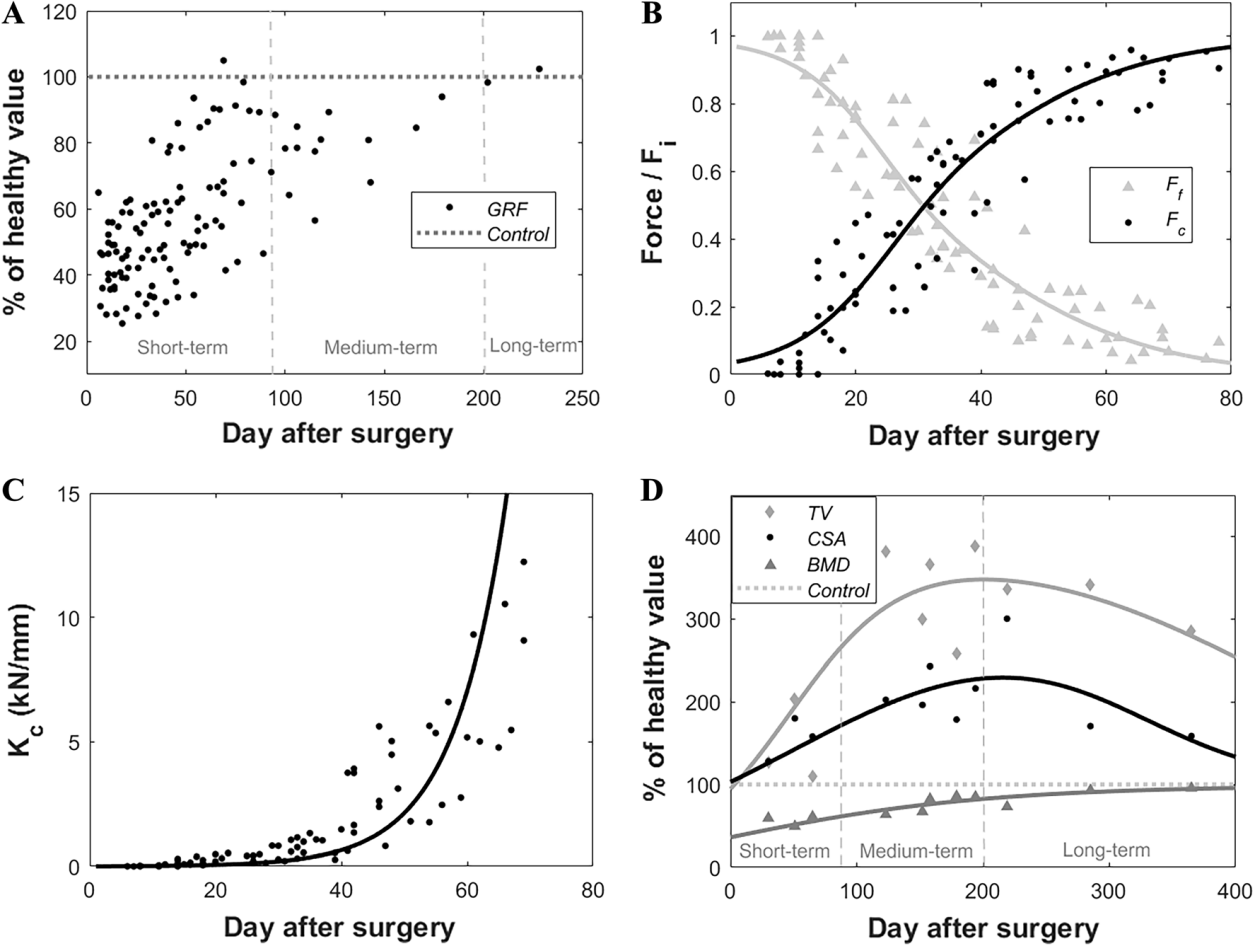
**A–D**: The evolution of the analyzed mechanical and imaging parameters over the regeneration time, including **A** the ground rection force ($$GRF$$) as a percentage of healthy values (dotted lines); the **B** fixation ($${F}_{f}$$) and callus force ($${F}_{c}$$); **C** the bone callus stiffness ($${K}_{C})$$; and **D** the bone callus total volume ($$TV$$), defect cross-sectional area ($$CSA$$), and bone callus mineral density ($$BMD$$), as a percentage of the healthy values (dotted line)

The evolution of analyzed gait parameters through the consolidation phase is shown in Fig. 5A. According to the $GRF$ data, TE surgery decreased the animals' bearing functional capacity in the operated limb at various levels. The week after the latency period, the stance phases generally reported a reaction force between 28 and 56% of healthy values (42–43% of the body weight in this animal model) [30, 33]. This gait parameter tended to a progressive recovery of approximately 56–85% at month 4 after surgery or up to 93% after half a year.

From the mechanical point of view, the ${F}_{i}$ distribution provided in Fig. 5B indicated an initial lack of mineralized connection between the scaffold and the original diaphysis. Thereby, the fixator loaded most of the physiological forces (${F}_{f}$ > 90% of ${F}_{i}$) during the first healing fortnight. From then on, the early bone calluses acquired greater mechanical relevance until the distribution of forces was overturned after an average of 30 consolidation days, thus being ${F}_{c}$ higher than ${F}_{f}$. In 70 days, the recovery of their bearing capacity was completed, and the forces through the external fixator stabilized around 0. At the same time, the bone calluses experienced a rapid exponential increase in their mechanical properties, as shown in Fig. 5C. While their mean apparent stiffness was quantified up to 650 N/mm on day 40 after surgery, they reached an average 7 kN/mm by 20 days later.

### Callus geometry and mineralization

**Fig. 6:**
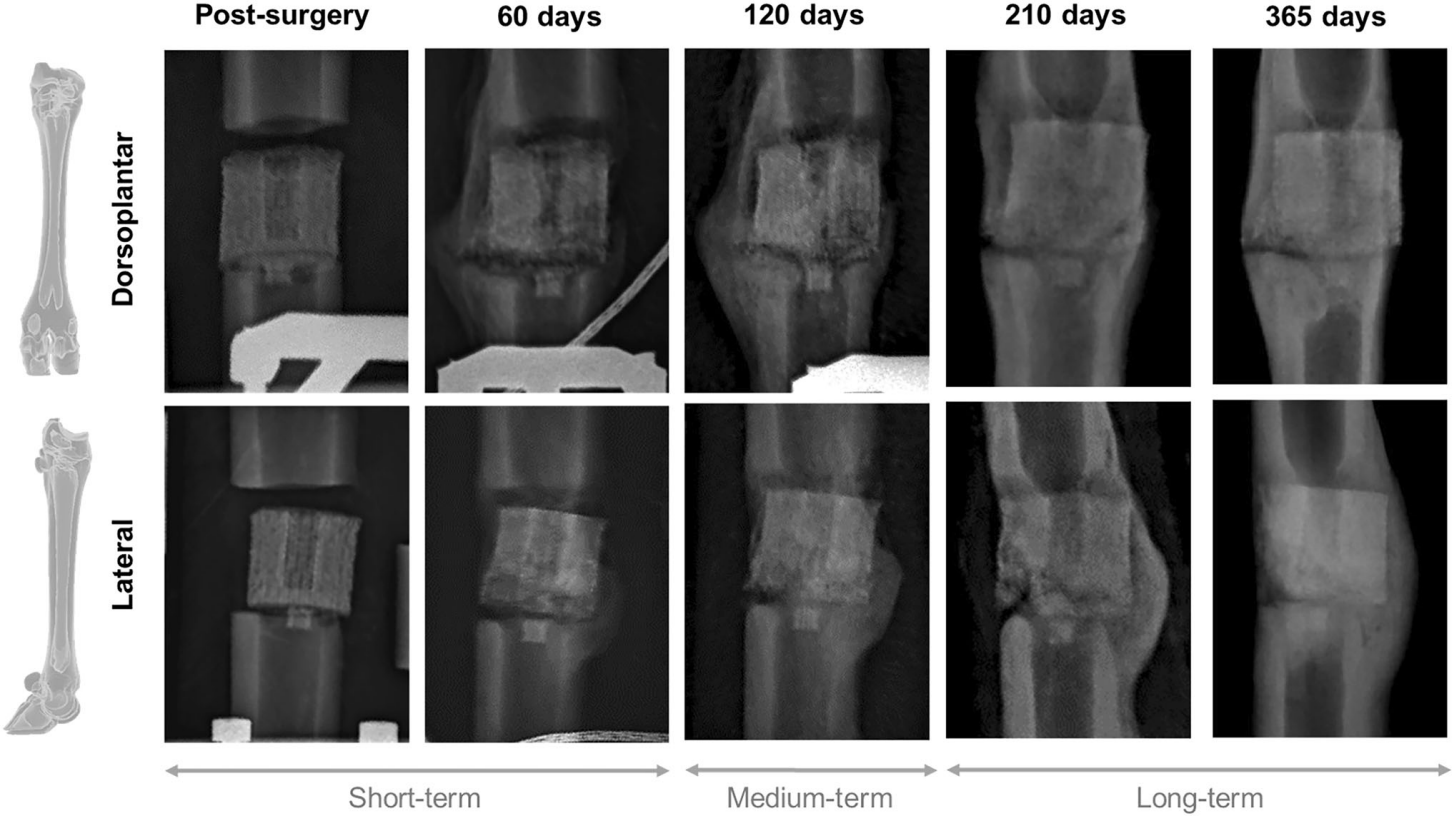
X-ray follow-up of the operated metatarsi at different time-points of the consolidation phase, days 0, 60, 120, 210, 365 days after surgery. First row corresponds to the dorsoplantar view and the second row to the lateral view of the imaging assessment in the same sheep

The progress of the mineralization in the same sheep at different time-points of the regeneration process is compared in Fig. 6. Post-surgical x-ray was included as a reference for the initial callus status. Despite the fact that the implant was clearly visible radiographically at any point of the experiment, it was impossible to appreciate the first stages of regeneration due to the ceramic opacity. Although the scaffold was designed to withstand the daily loads of the animal, it was logistically impossible to ensure the absence of instantaneous peaks of extreme loads due to unexpected movements or behavior. For example, the lateral views in Fig. 6 show a local fracture in the distal left corner of the implant in the images obtained at days 120 and 210 after surgery. These minor breakages occurred in approximately half of the specimens in line with what occurred in similar studies [20]. Fortunately, they did not affect the general integrity of the structure. In more advanced stages of the consolidation phase, ossification around the scaffold was generally observed, especially in the posterior interzone, where physiologically more significant vascularization is found due to the location of the flexor tendons. Signs of woven bone remodeling in the global callus geometry were also reported after one year of regeneration.

The evolution of the parameters quantified by CT imaging are shown in Fig. 5D, including the total volume ($TV$), the defect cross-sectional area ($CSA$), and the bone mineral density ($BMD$). They are presented as a percentage of their 15 mm non-treated contralateral value, being 2.17 ± 0.26 cm^3^, 144.79 ± 14.42 mm^2^, 1.25 ± 0.07 g HA/cm^3^, respectively. Both $TV$ and $CSA$ start from values close to healthy values due to the subject-specific nature of the implant. However, according to the fitting function, both parameters rapidly increase throughout the first half of the year. The maximum peak values were reported after approximately 200 days from surgery, averaging 347.88% of the control value for $TV$ and 229.49% for $CSA$. During the second half of the year, there is a remodeling slightly tending to healthy values. After one year, the $TV$ and $CSA$ were still 280.82% and 156.13% over control. For its part, the $BMD$ was initially 36% of the healthy mineral density due to the apparent mineral content of the scaffold, which was measured individually with the same CT scan. This parameter experienced a continuous recovery of up to 95% of the contralateral value at the end of the experimental year.

## Discussion

The current work provides an interdisciplinary characterization of the TE callus consolidation throughout the evolution of different mechanical and imaging parameters measured *in vivo* and *ex vivo*. The overall thrust was towards a gradual recovery of their healthy conditions by means of a flexible fixation and subject-specific hydroxyapatite scaffolds that remained structurally sturdy throughout the entire process. Specifically, the surgical methodology of leaving an appropriate gap between the brittle porous substitute and the proximal loading fragment, filled with spongy tissue, successfully prevented fatal stress concentrations via local contact. Nor was it required to resort to bone morphogenetic proteins or other bioactive molecules to support and choreograph bone tissue formation. Thus, the optimized scaffold seems to meet by itself the clinical requirements of mechanical support and osteoinductivity, and validates *in vivo* the numerical optimization of its microstructure [40]. This research offers uncomplicated strategies to investigate further the combination of bioceramic materials and instrumented external fixators, as well as the need or not to biologically vitaminize the scaffold with growth factors or other agents that induce regeneration.

The healing of the monitored parameters showed different speeds. In the short term (0–3 months after surgery), a bone callus began to grow between the diaphysis and the scaffold, as shown in Fig. 6 (post-surgery and day 60). This was reflected in a clear recovery of the mechanical functionality, loading capacity and apparent stiffness of the defect (see Fig. 5B, C). Compared to other bone regeneration processes in the same animal model [9, 30, 31], the natural stiffness of the implant contributed to faster mechanical governance of the internal force distribution. For instance, the externally stabilized 15 mm bone transport calluses of Mora-Macías et al. [9] needed an average of 10 additional days to overreach the fixator’s loading (${F}_{f}<{F}_{c}$) in the same bone model. Moreover, while bone transport defects recovered completely their capacity of sustaining the metatarsal force (${F}_{c}$ ~ 80–90% of the ${F}_{i}$) after 70 days from surgery, TE achieves it in just 50 days. A similar healing advance is found when comparing with a 15 mm lengthening callus Blázquez-Carmona et al. [30] using the same fixation employed in the current study. For its part, Grasa et al. [31] quantified a stabilization of the forces through their unilateral fixator in 30 consolidation days. Nevertheless, they mechanically investigated the healing of a shorter and more vascularized 2 mm tibial fracture. Regarding the apparent callus stiffness ${K}_{C}$, TE callus also presented a more exponential behavior than the distraction alternatives by reaching stiffness quotes over 15 kN/mm in less than 70 days after surgery. In the same period, the distraction calluses of Blázquez-Carmona et al. [30] and Mora-Macías et al. [4, 45] did not even reach half of that stiffness. However, the calibration limits of the measurement equipment prevented us from exploring if the inclusion of the ceramic scaffold alters the predictable stabilization around the stiffness of an intact metatarsus in the short term [46].

In the medium term (3–7 months after surgery), once the naïve bridging callus ended mineralizing (Fig. 6, days 120 and 210), $GRF$ returned to healthy conditions after the undeniable surgical effect (see Fig. 5A). This recovery was reported to be shorter in other bone regeneration processes. For instance, Mora-Macías et al. [33] quantified approximately healthy $GRF$ values after about 3–4 months after surgery owing to a less pronounced lameness in an equivalent metatarsal defect treated by bone transport: a loss between 0 to 10% of the $GRF$ in the distraction group vs. the 60% in TE. An intermediate initial lameness was obtained in the ovine fracture healing study of Seebeck et al. [47]. They quantified the $GRF$ in 70% of the control value after distracting a 3 mm tibial gap. The significant TE lameness could be associated with a more prolonged lack of bone continuity, especially compared to fracture healing approaches. In bone transport, once distracted, the hematoma formed during the latency phase ensures an early connective template between the distal and proximal fragments [8, 48]. Consequently, TE animals could instinctively develop gait compensation mechanisms by relying on non-operated limbs.

**Fig. 7:**
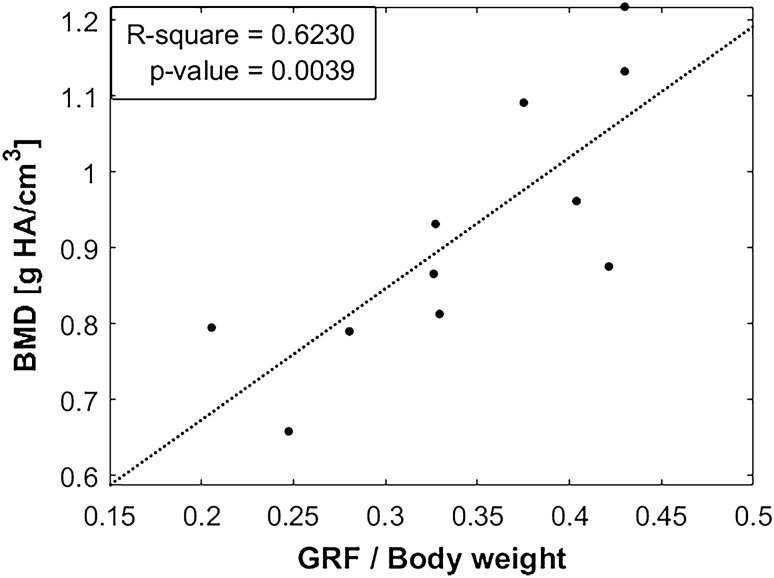
Linear correlation between the ground reaction force ($GRF$, normalized by the body weight of the animals) and the bone mineral density ($BMD$)

The progressive recovery of the gait parameter seemed to shear similar upward behavior to that experienced by $BMD$, being over 80% of the cortical mineral density after six months (see Fig. 5D). In the literature, Pobloth et al. [38] measured the same parameter in their 40 mm ovine tibia gaps. After 12 weeks, they found a mineral degree of 0.65 g HA/cm^3^ through CT images and 0.76 g HA/cm^3^ when using micro-CT. A slightly higher $BMD$ was estimated at close time-points in our animals, being 0.79 g HA/cm^3^ at day 65 after surgery and 0.81 g HA/cm^3^ at day 123, possibly due to the smaller defect size to regenerate. Li et al. [49] also quantified the mineral content in 5 mm rodent tibia defect replaced by a lithium structure enhanced with mesenchymal stem cells and deproteinized bone tissues. After 8 weeks, a non-grafted group presented a $BMD$ of 0.41 g HA/cm^3^, being upgraded to 0.57–0.87 g HA/cm^3^ in the biologically enhanced scaffolds. According to the correlation presented in Fig. 5D, our bone callus would have a 0.55 g HA/cm^3^ mineral content at this consolidation stage. The interdifferences between animal and bone models would explain the small dissimilarities. Returning to the similar trend between $GRF$ and $BMD$, Fig. 7 builds a linear regression between these parameters to evaluate possible significant correlations. Assuming a significance with R-square > 0.5 and *p*-value < 0.005, it is deduced that the recovery of the mobility disorders developed by animals could be subjected to complete callus mineralization. Probably overcoming this ossification stage, the subjects definitely regained confidence in their operated limb.

Finally, in the long term (> 7 months), it was observed the progressive structural reshaping of the consolidation callus until the original geometry (Fig. 6, day 365). This effect is materialized through the evolution of the callus $TV$ and $CSA$. As mentioned above, the time point at which the transition from the consolidation phase to the remodeling phase occurs approximately 200 days after surgery when a reduction in the volume of the surrounding callus began to be quantified. This represents a delay of about 80 days with respect to the bone transport alternative in the same bone model and defect [50]. Nonetheless, the maximum volume reached is almost identical in both treatments, finding around 7.60 cm^3^ of woven bone filling a 15 mm metatarsal defect, around 0.37 cm^3^ per longitudinal millimeter of the metatarsal defect. Comparing with other ovine bone tissue engineering studies, Vidal et al. [20] only reported around 50% of the original bone volume of a 35 mm metatarsal defect after 3 consolidation months, which could be explained by the use of a less mechanically stimulating internal fixation. Regarding other studies with external solutions, Pobloth et al. [38] also showed a less solid 40 mm bony union consistency 12 weeks after surgery. At this time-point, they reported a 0.043 and 0.067 cm^3^ of regenerated bone per millimeter of tibial defect treated with their ceramic scaffold without and with an additional autologous, respectively. These differences are even more remarkable by considering the different bone models used in each study since distal bones are characterized by a lower bone repair capability compared to proximal ones in tissue engineering [51]. The only explanation would again be differences in the defect size.

In conclusion, the combination of grafted bioceramic scaffolds and flexible external fixators represents an effective approach to sustain bone formation and osseointegration in segmental critical-size load-bearing defects. The regeneration process is characterized by faster recovery of the bone callus mechanical function but with a slower normalization of the subject's walking conditions compared to other orthopedic alternatives, which seems to be related to the total bone mineral density healing. Despite these differences in recovery speed, all the analyzed structural and mechanical parameters progressed toward control values without setbacks, similar to other bone regeneration processes [30, 31, 33, 48]. Consequently, the bone function is suggested not to be altered at any regeneration stage by the scaffold osseointegration or by the minor local breakages of the structure. Prospectively, the designed methodology opens the clinical doors to a valuable continuous mechanical follow-up of the entire bone regeneration process, far from the usual *ex vivo* analyses at discrete time-points after the specimen’s sacrifice [18–20, 38]. Thus, these mixed mechanical and imaging monitoring techniques are applicable to any bone tissue engineering experiments with any biomaterial and 3d-printed microarchitecture. Comparisons in the speed of recovery of the parameters analyzed could serve as the basis for optimizing novel tissue engineering substitutes and orthopedic approaches in the field.
